# Supplementary material for: Detection of a sequence feature for recursive splicing
Source: bioRxiv. 2026 Apr 17:2026.04.13.717821. Preprint. [Version 1] doi: 10.64898/2026.04.13.717821 (PMC13105047; doi:10.64898/2026.04.13.717821)
Supplement: Supplement 1 [file NIHPP2026.04.13.717821v1-supplement-1.pdf]

bioRxiv preprint doi: https://doi.org/10.64898/2026.04.13.717821; this version posted April 17, 2026. The copyright holder for this preprint (which was not certified by peer review) is the author/funder. This article is a US Government work. It is not subject to copyright under 17 USC 105 and is also made available for use under a CC0 license.

Supplementary Figures

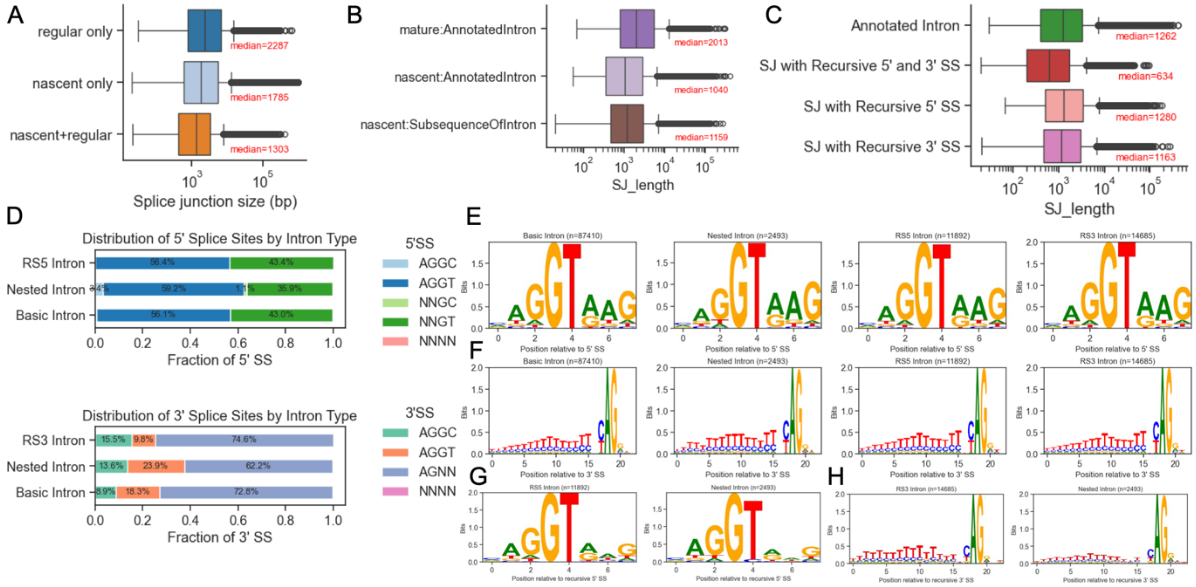

Supplementary Figure 1. Nascent RNA-Seq revealed 4 types of introns.

A. Distribution of unique splice junctions in either nascent or regular RNA-Seq and those ubiquitously used in both data.
B. Distribution of lengths of splice junctions uniquely expressed in either nascent or regular RNA-Seq.
C. Distribution of splice junction lengths in 4 types of introns.
D. Fractions of 5’SS tetramers and 3’SS tetramers at recursive splice sites.
E. Sequence logo of annotated 5’SS of 4 types of introns.
F. Sequence logo of annotated 3’SS of 4 types of introns.
G. Sequence logo of recursive 5’SS of 2 types of introns.
H. Sequence logo of recursive 3’SS of 2 types of introns.

bioRxiv preprint doi: https://doi.org/10.64898/2026.04.13.717821; this version posted April 17, 2026. The copyright holder for this preprint (which was not certified by peer review) is the author/funder. This article is a US Government work. It is not subject to copyright under 17 USC 105 and is also made available for use under a CC0 license.

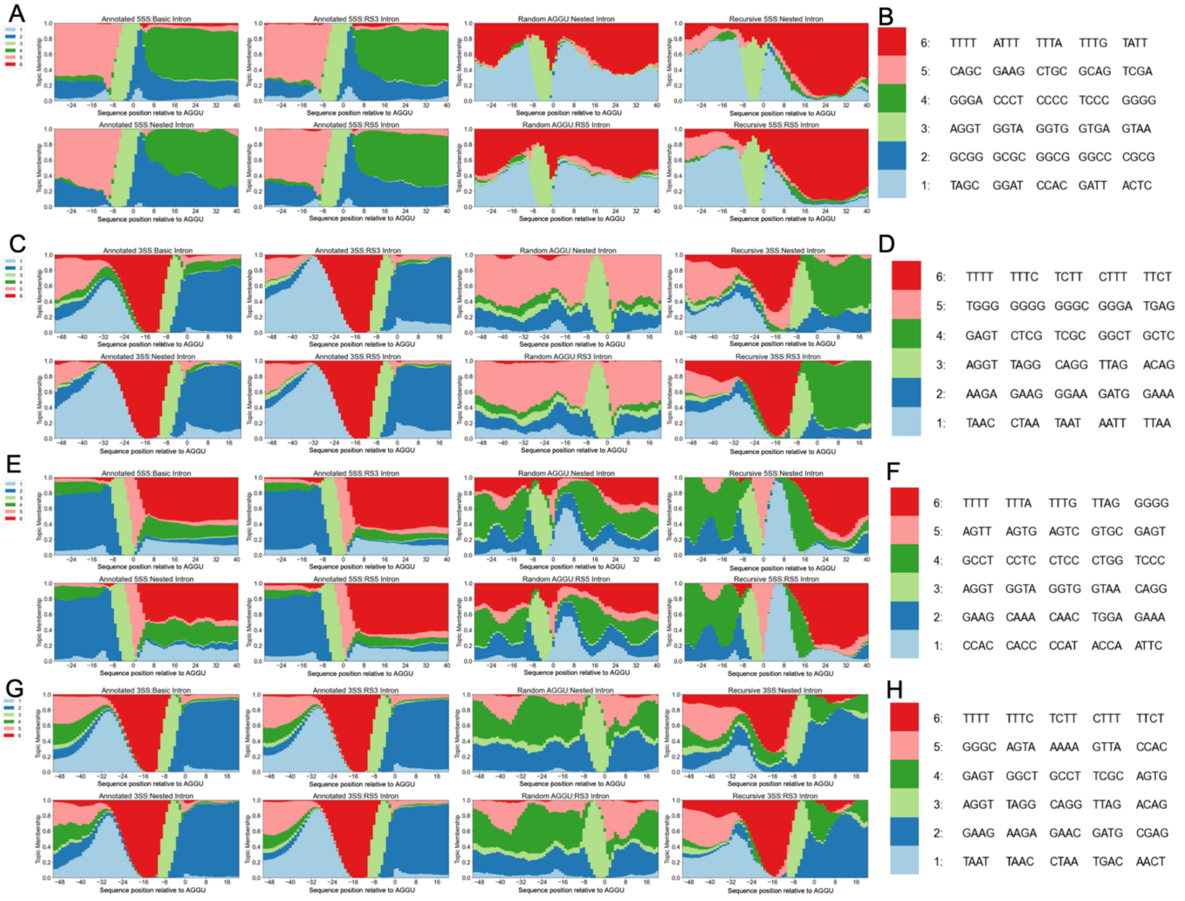

Supplementary Figure 2. Mixture models uncovered potential cis-acting factors of recursive splicing.

A. Structure plot of topic/cluster distribution of regions from 30bp upstream to downstream 50bp of canonical and recursive 5'SS in the first introns. Random AGGU sites were also shown.
B. Top ranked k-mers in 5'SS model in A.
C. Structure plot of topic/cluster distribution of regions from 50bp upstream to 30bp downstream of 3'SS in the first introns.
D. Top ranked k-mers in 3'SS model in C
E. Structure plot of topic/cluster distribution of regions from 30bp upstream to downstream 50bp of canonical and recursive 5'SS in the downstream introns. Random AGGU sites were also shown.
F. Top ranked k-mers in 5'SS model in E.
G. Structure plot of topic/cluster distribution of regions from 50bp upstream to 30bp downstream of 3'SS in the downstream introns.
H. Top ranked k-mers in 3'SS model in G.

bioRxiv preprint doi: https://doi.org/10.64898/2026.04.13.717821; this version posted April 17, 2026. The copyright holder for this preprint (which was not certified by peer review) is the author/funder. This article is a US Government work. It is not subject to copyright under 17 USC 105 and is also made available for use under a CC0 license.

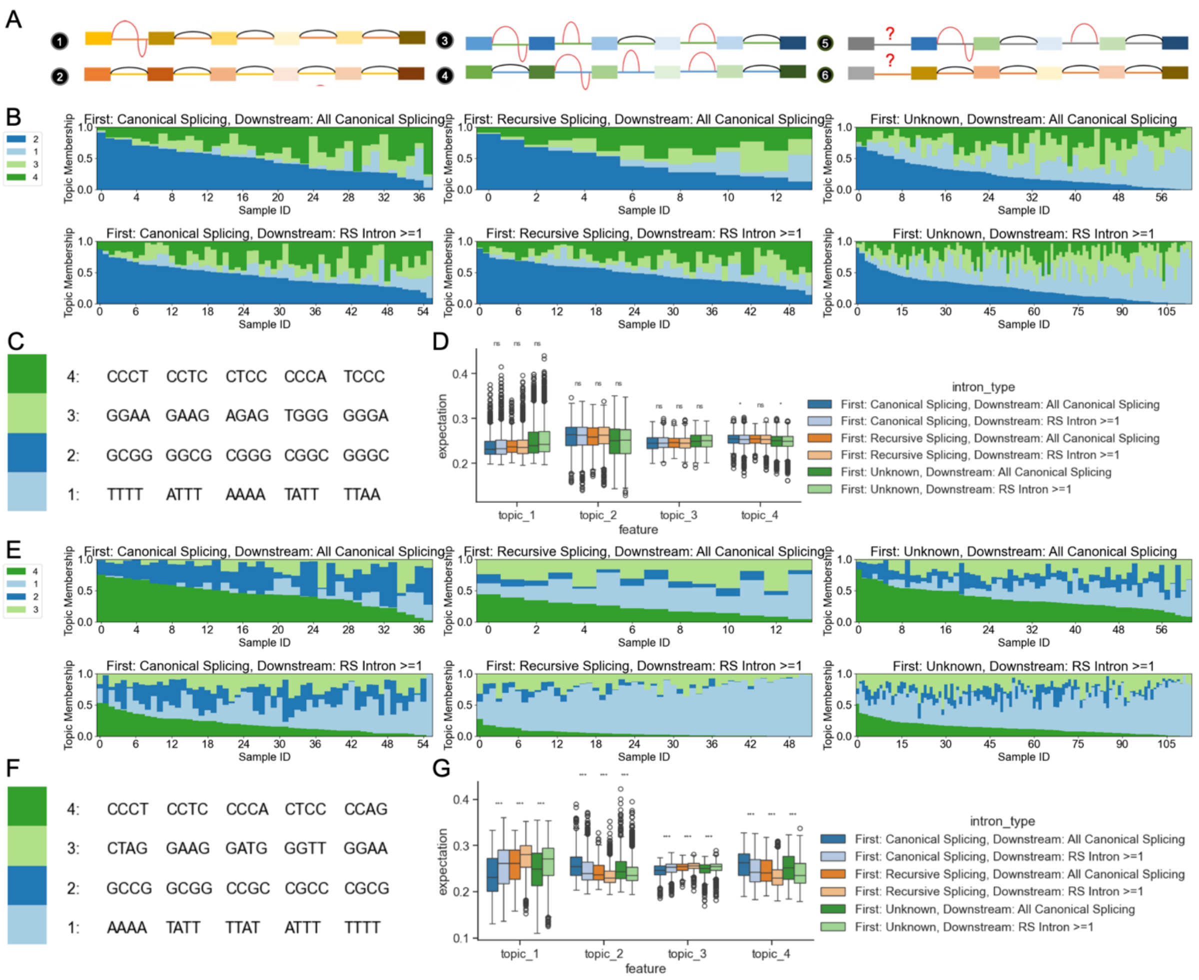

Supplementary Figure 3. Sequences of the annotated 5’SS and 3’SS of the first intron indicate recursive splicing site usage.

A. Diagram of 6 types of transcription units based on the splicing status in the first and downstream introns.
B. Structure plot of topic/cluster distribution of regions from 50bp upstream to 50bp downstream of the canonical 5’SS in the first introns.
C. Top ranked k-mers in 5’SS model in B.
D. Distribution of single sequence topic expectation values calculated with model B.
E. Structure plot of topic/cluster distribution of regions from 50bp upstream to 50bp downstream of the canonical 3’SS in the first introns.
F. Top ranked k-mers in 3’SS model in E.
G. Distribution of single sequence topic expectation values calculated with model E.

bioRxiv preprint doi: https://doi.org/10.64898/2026.04.13.717821; this version posted April 17, 2026. The copyright holder for this preprint (which was not certified by peer review) is the author/funder. This article is a US Government work. It is not subject to copyright under 17 USC 105 and is also made available for use under a CC0 license.

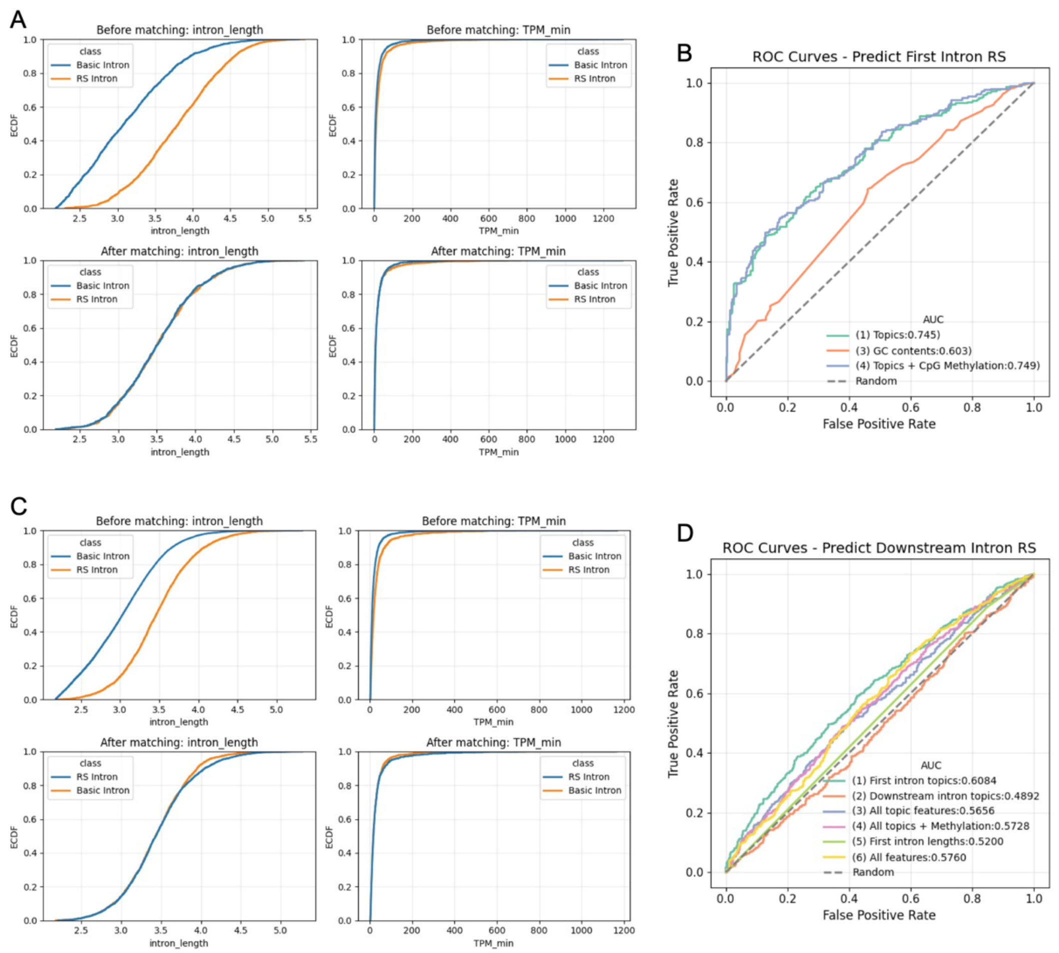

Supplementary Figure 4. Computational modeling of sequence features aids in the accurate prediction of recursive splicing.

A. Cumulative distribution of intron length and TPM of basic and RS introns (first introns of genes) before and after adjusting matching sets. To evaluate the the performance without the impact of intron length or gene expression, we select subsets of basic and RS first introns with similar length and TPM distribution.
B. ROC curve of predicting RS in first introns. Mixture model topics and DNA methylation can help predict RS with accuracy of 74.9%
C. Cumulative distribution of intron length and TPM of basic and RS introns (downstream introns of genes) before and after adjusting matching sets.
D. ROC curve of predicting RS in downstream introns. Sequence features in the corresponding first introns help predict RS in downstream introns of the same genes with an accuracy of 60.8%.

bioRxiv preprint doi: https://doi.org/10.64898/2026.04.13.717821; this version posted April 17, 2026. The copyright holder for this preprint (which was not certified by peer review) is the author/funder. This article is a US Government work. It is not subject to copyright under 17 USC 105 and is also made available for use under a CC0 license.

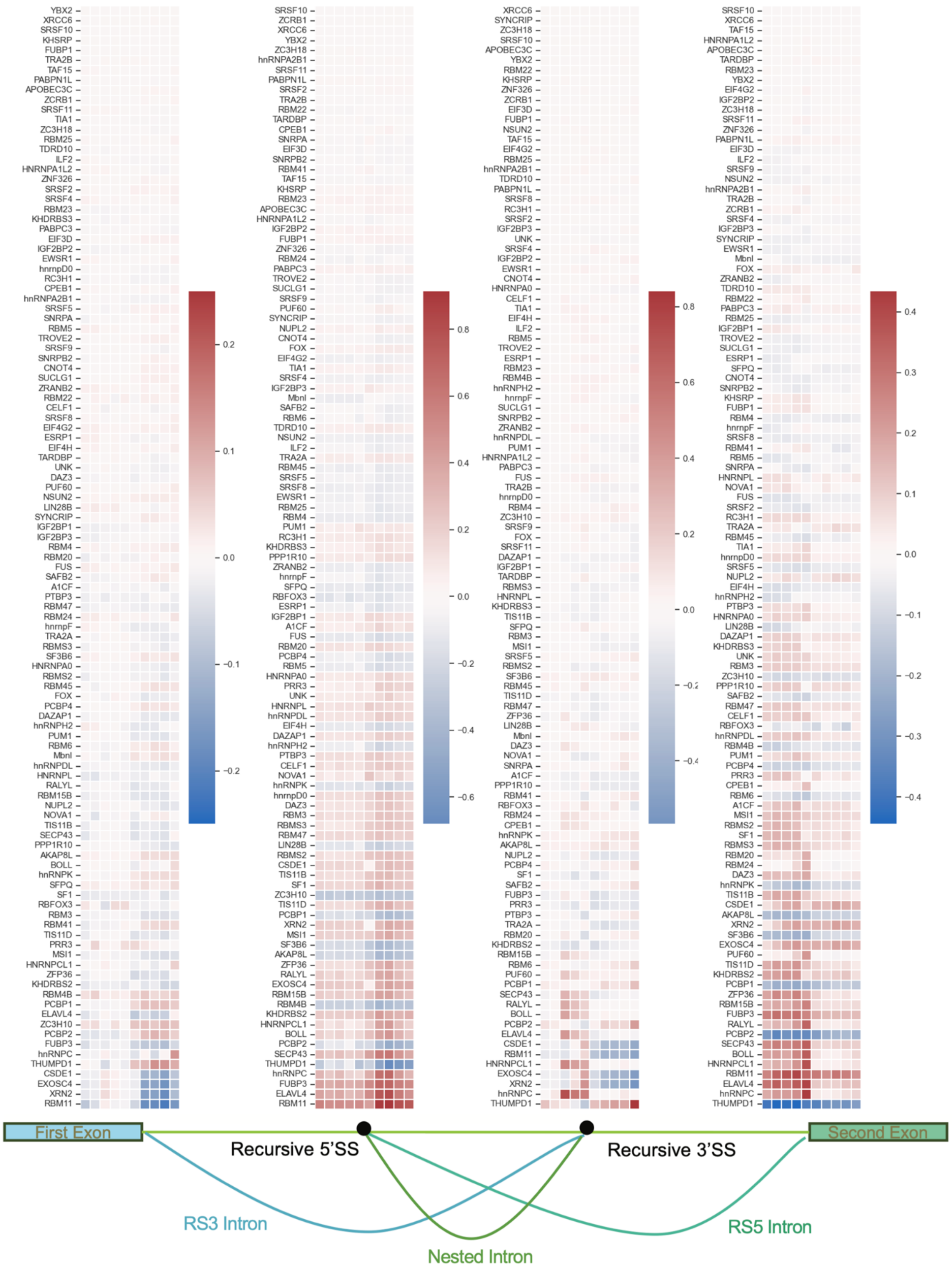

Supplementary Figure 5. In vitro protein binding assay uncovers potential trans acting RBPs regulating recursive splicing.
Full heatmaps of the L2FC of protein-binding enrichment scores in F7B.
